# Dynamic Prognostication in Transplant Candidates with Acute-on-Chronic Liver Failure

**DOI:** 10.3390/jpm10040230

**Published:** 2020-11-15

**Authors:** Cheng-Yueh Lu, Chi-Ling Chen, Cheng-Maw Ho, Chih-Yang Hsiao, Yao-Ming Wu, Ming-Chih Ho, Po-Huang Lee, Rey-Heng Hu

**Affiliations:** 1 Department of Surgery, National Taiwan University Hospital and College of Medicine, Taipei 100, Taiwan; lusmalleye0420@gmail.com (C.-Y.L.); cyhsiao1102@gmail.com (C.-Y.H.); wyaoming@gmail.com (Y.-M.W.); mcho1215@ntu.edu.tw (M.-C.H.); pohuang1115@ntu.edu.tw (P.-H.L.); rhhu@ntu.edu.tw (R.-H.H.); 2 Graduate Institute of Clinical Medicine, National Taiwan University, Taipei 100, Taiwan; chlnchen@ntu.edu.tw; 3 Department of Traumatology, National Taiwan University Hospital, Taipei 100, Taiwan; 4 Department of Surgery, E-Da Hospital, I-Shou University, Kaohsiung 886, Taiwan

**Keywords:** acute-on-chronic liver failure, prognosis, liver transplant, dynamic risk factor

## Abstract

We aimed to extensively investigate clinical markers that are sufficiently dynamic for prognosis of acute-on-chronic liver failure (ACLF). Defined by the Asian Pacific Association for the Study of the Liver (APASL) criteria, patients with ACLF on the liver transplant waitlist in a tertiary center were retrospectively reviewed. Laboratory results and severity scores at three time points (days 1, 7, and 14 after admission) were analyzed. From 2015 to 2019, 64 patients with ACLF were enrolled, of which 24 received a liver transplant from 22 live donors. The hospital mortality rate was 31% (8% for transplant; 45% for nontransplant groups), and the 3-month survival was crucial for determining long-term outcomes. The number of significant variables for mortality, and, specifically, the hazards of international normalized ratio of prothrombin time (INR) and APASL ACLF Research Consortium (AARC) score were increased within two weeks. In multivariable analysis, INR and AARC score (D-14) were associated with poor survival and liver transplant was a protective factor in all patients, while AARC score (D-14) was significant in the nontransplant group. AARC score at day 14 is an independent risk factor for mortality in ACLF. Liver transplant from live donors reversed poor outcomes in patients with ACLF in a timely manner.

## 1. Introduction

Acute-on-chronic liver failure (ACLF) is a clinical syndrome manifesting as acute and severe hepatic dysfunction in patients with chronic liver disease caused by various insults [1]. Acute precipitants include infection (systemic nonviral infection or via hepatotropic viruses), toxins (alcohol or drugs), and bleeding, whereas the underlying chronic liver disease (generally cirrhosis) can be due to hepatitis B or C virus (HBV or HCV) infection, alcohol, or nonalcoholic steatohepatitis or be of autoimmune or cryptogenic origin [1,2,3]. The prevalence of these factors vary greatly by geography [2]. Permutations and combinations of known and/or unknown etiologies have led to heterogeneous ACLF presentation and regional differences and inconsistent diagnostic criteria [3]. Spectrum heterogeneity influences patient prognosis, although all patients with ACLF have high short-term mortality [4]. Therefore, this important issue warrants investigation, and experiences from centers worldwide should be evaluated [2].

Furthermore, even with the same order and combination of etiologies, patients with ACLF may have a variable disease course due to different acute immunoinflammatory responses [3] and functional liver reserves. This contributes to the highly dynamic nature of the course of ACLF and makes early prognostication and triage (regenerative recovery vs. expeditious liver transplant) a challenge [5]. Diagnostic criteria that adopt prognostic insights were inconsistent and not one-size-fits-all. For example, unlike the other definitions (the European Association for the Study of the Liver–chronic liver failure (EASL-CLIF) or the North American Consortium for the Study of End-Stage Liver Disease (NACSELD)), the definition of the Asian Pacific Association for the Study of the Liver (APASL) does not include extrahepatic organ failures [3]. Besides, cirrhotic liver background and acute decompensation by bacterial infection are essential diagnostic components in EASL-CLIF’s and NACSELD’s versions, but are not necessary in APASL’s version of the ACLF definition [4,6,7]. This controversy highlights the urgent need to agree on the one universal definition. Studies have attempted to identify prognostic markers that reflect the dynamic nature of ACLF; however, they have been scarce and inconclusive [4,8,9,10]. The aim of this study was to investigate clinically measurable factors that can dynamically reflect prognosis in a retrospective hospital ACLF cohort.

## 2. Methods

The Institutional Review Board of National Taiwan University Hospital, Taipei, Taiwan, approved this study (NTUH REC: 202004053RINB). Because this is a retrospective study based on chart review, the institutional review board waived the need for informed consent.

### 2.1. Patients

We reviewed the medical records of hospitalized patients who were registered as candidates for liver transplant on the waiting list of the Taiwan Organ Registry and Sharing Center from January 2015 to October 2019. Adult patients who fulfilled the ACLF diagnosis criteria were included. The diagnosis of ACLF was based on the criteria formalized by the ACLF consensus recommendations of the Asian Pacific Association for the Study of the Liver (APASL), defined as the presence of acute hepatic insult, jaundice (bilirubin ≥5 mg/dL), and coagulopathy (international normalized ratio (INR) ≥1.5) complicated by ascites or encephalopathy or both within 4 weeks, with previously diagnosed or undiagnosed chronic liver disease [6,11,12]. The index date was the date of admission when liver transplant evaluation was performed. Patients with malignancy and congenital diseases were excluded.

### 2.2. Demographic Parameters

Demographic information including sex; age; body mass index; comorbidity, such as hepatitis B virus (HBV), hepatitis C virus (HCV), cirrhosis, diabetes mellitus (DM), hypertension, dyslipidemia, autoimmune diseases, coronary arterial disease, or chronic kidney disease; and clinical laboratory variables at the 1st, 7th, and 14th day of hospital stay were collected. Laboratory data after liver transplant when the patients received transplant surgery within 2 weeks after admission were excluded. The severity of liver disease was assessed using the APASL ACLF Research Consortium (AARC) score [6] and Model for End-Stage Liver Disease (MELD) score [13]. The date of liver transplantation was recorded.

### 2.3. Outcome Measurement

The patients were followed up until death or 31 January 2020. All patients were followed up for at least 3 months. The event date was the date of death or the last follow-up date.

### 2.4. Statistical Analysis

Descriptive statistics are expressed as mean ± standard deviation or number (percentage) when appropriate. Variables were compared using a Student’s *t*-test, χ^2^ test, or Fisher’s exact test. Cumulative survival rates were estimated using the Kaplan–Meier method and compared using the log-rank test. Cox’s proportional hazard model was used for univariable and multivariable analyses. The results were statistically significant when the two-sided *p* value was <0.05. Analyses were performed using SPSS version 21.0 (IBM Corporation, Armonk, NY, USA).

## 3. Results

### 3.1. Demographics

**Figure 1:**
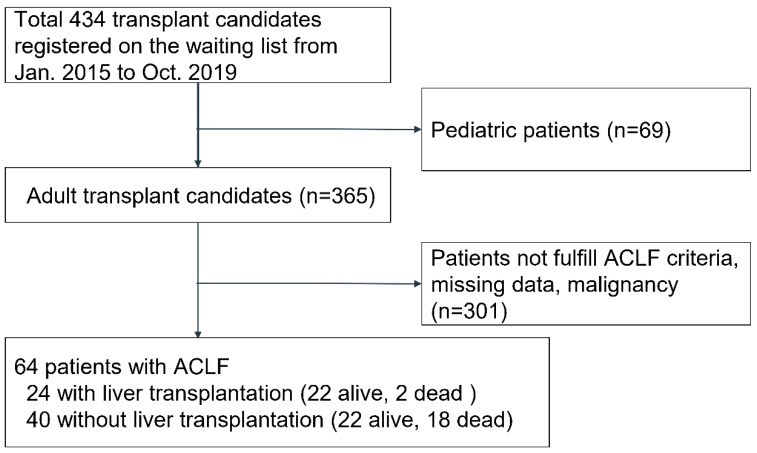
Schematic of the patient selection process.

During the study, 434 patients, including 365 adults, were identified on the waiting list (Figure 1). A total of 64 (17.5%) patients met the ACLF diagnostic criteria and were included in the analysis. Most patients were men (46, 71.9%), with HBV-associated etiologies (56, 87.5%) and an average age of 53.5 ± 9.9 years. Other etiologies were drug induced (1), autoimmune related (1), hemolysis, elevated liver enzyme, and low platelet (HELLP) syndrome related (1), and unknown (5). All HBV patients were commenced on entecavir or tenofovir or both. No HCV patients were in this cohort. The mean follow-up time was 16.9 months. Subsequently, 24 (37.5%) patients received a liver transplant (22 live and two deceased donors) after a mean waiting time of 27 days. A total of seven patients had grade III or IV hepatic encephalopathy. One patient with grade IV encephalopathy, who was referred from another hospital, was intubated and received plasma exchange and a subsequent liver transplant within 1 week after admission. The hospital mortality was 31% for all patients, 45% for patients without transplant, and 8% for patients with transplant.

The demographic and laboratory characteristics are shown in Table 1. Compared with the nontransplant group, the transplant group had a higher percentage of those with HBV (100.0% vs. 80.0%, *p* = 0.038), cirrhosis (95.8% vs. 10.0%, *p* < 0.001), higher degree of ascites (*p* < 0.001), plasma exchange (41.7% vs. 12.5%, *p* = 0.014) and a transfer from other hospitals (58.3% vs. 27.5%, *p* = 0.033). The transplant group had a higher white blood cell (WBC) count at day 7 (D-7) (9.4 vs. 7.6 × 10^3^/μL, *p* = 0.049) and creatinine at day 14 (D-14) (2.1 vs. 1.1 mg/dL, *p* = 0.009) and lower platelet count at day 14 (D-14) (91.3 vs. 145.3 × 10^3^/μL, *p* = 0.002). Average MELD and AARC scores in 64 patients at days 1, 7, and 14 were 26.3 ± 12.6, 29.6 ± 15.5, and 30.2 ± 16.7; 8.4 ± 2.3, 8.6 ± 1.9, and 9.0 ± 2.2, respectively. Both MELD and AARC scores were not significantly different between the two groups.

The demographic and laboratory characteristics of the nontransplant patients were further stratified by 3-month mortality, as shown in Table 2. Compared with the non-survival group, the recovery group had a younger age (50.0 vs. 59.6 years, *p* = 0.006), a lower percentage of those with HBV (68.2% vs. 100.0%, *p* = 0.011), less cirrhosis (18.1% vs. 50.0%, *p* = 0.039), a lower degree of ascites (*p* = 0.006) and plasma exchange (0% vs. 27.8%, *p* = 0.011), lower MELD score D-7 (22.8 vs. 34.2, *p* = 0.014) and D-14 (20.9 vs. 37.1, *p* = 0.002), AARC score D-7 (7.8 vs. 9.2, *p* = 0.028) and D-14 (7.5 vs. 10.3, *p* < 0.001), INR D-7 (1.69 vs. 3.02, *p* = 0.011) and D-14 (1.6 vs. 3.3, *p* < 0.001), sodium D-14 (134.2 vs. 139.0 mmol/L, *p* = 0.025), creatinine D-14 (0.9 vs. 1.4 mg/dL, *p* = 0.006), and ammonia-D14 (65.1 vs. 85.8 μmol/L, *p* = 0.039), and higher platelet count D-7 (159.5 vs. 110.8 × 10^3^/μL, *p* = 0.030) and D-14 (178.6 vs. 99.5 × 10^3^/μL, *p* < 0.001).

### 3.2. Overall Survival

**Figure 2:**
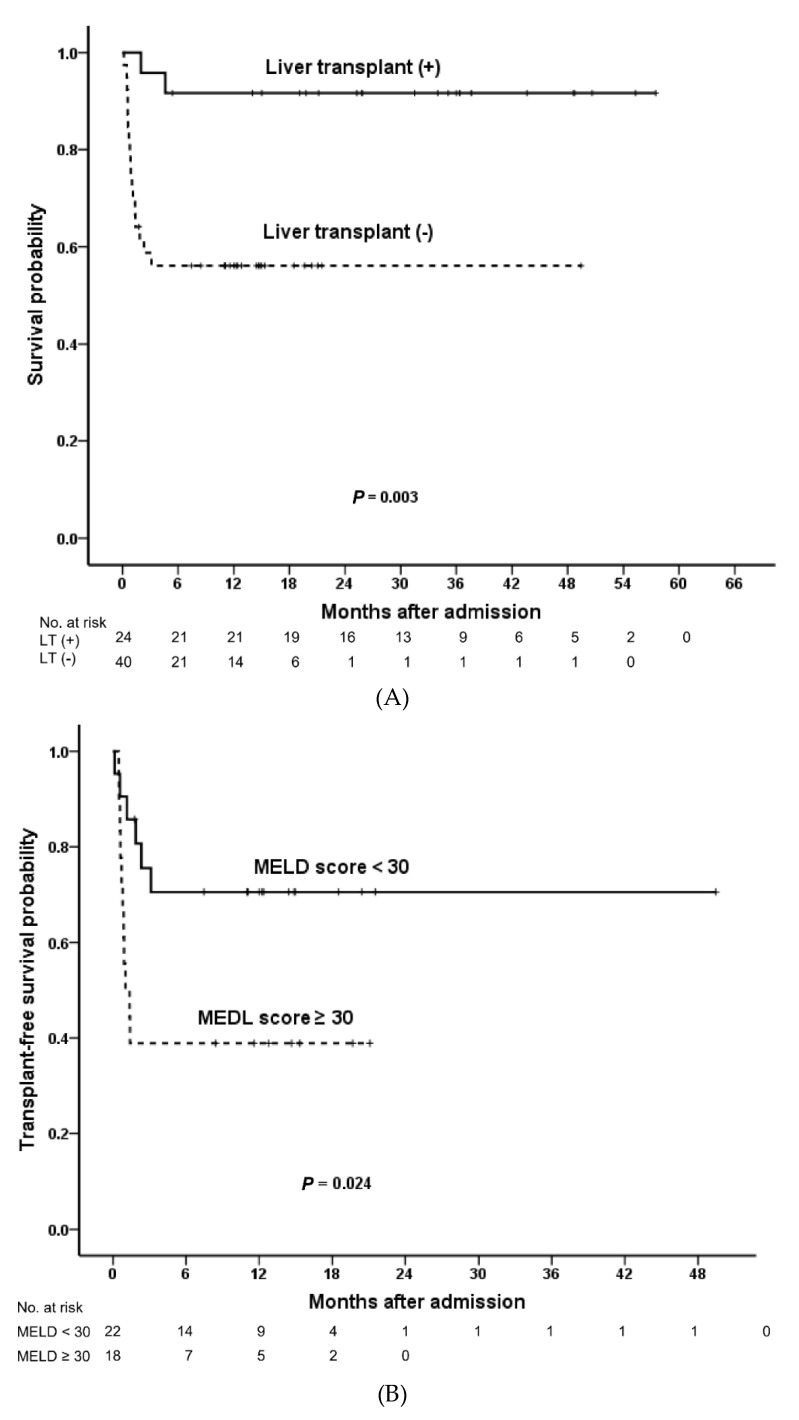

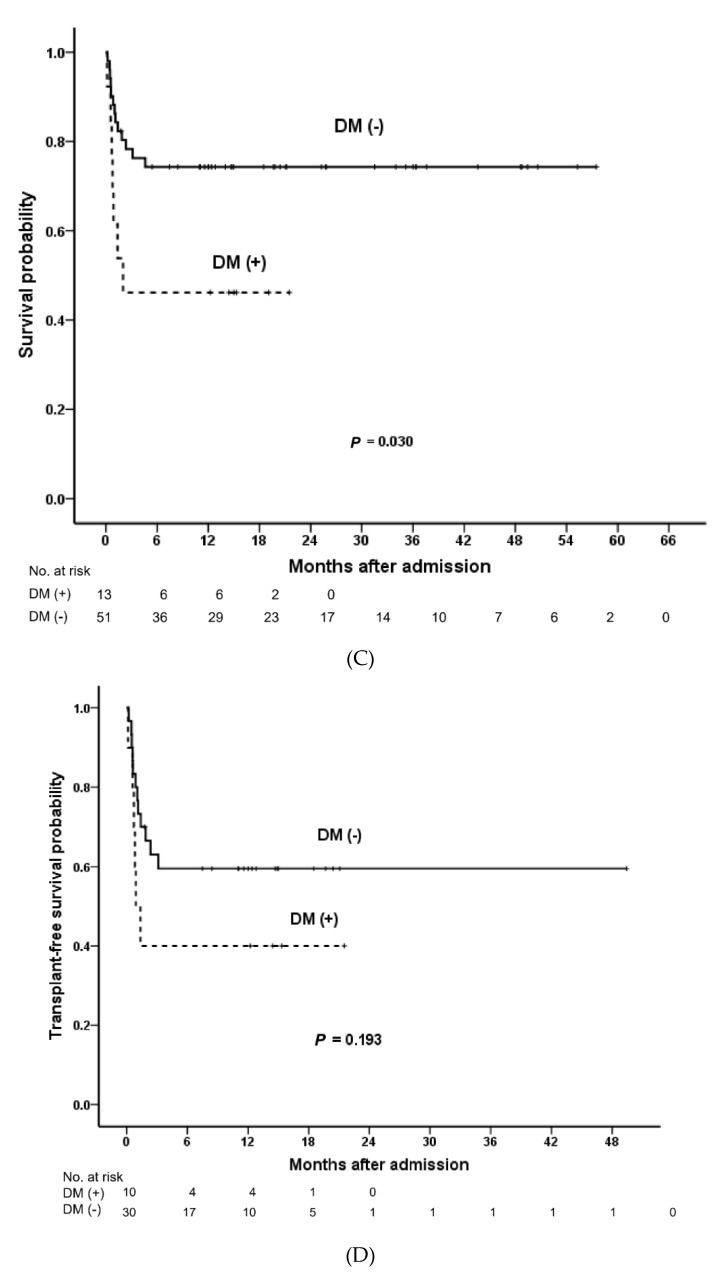
Overall survival in patients with acute-on-chronic liver failure. (**A**) Patients who received liver transplant vs. those who did not. (**B**) MELD score ≥30 vs. <30 in subgroup patients without transplant. Survival stratified based on the presence of DM in all patients (**C**) and the nontransplant group (**D**) DM, diabetes mellitus; MELD, Model for End-stage Liver Disease.

The 1-month, 3-month, 6-month, 1-year, and 3-year survival rates were 95.8%, 91.7%, 91.7%, 91.7%, and 91.7%, respectively, in the transplant group and 74.4%, 56.1%, 56.1%, 56.1%, and 56.1%, respectively, in the nontransplant group (Figure 2A). Patients survived, irrespective of a transplant, if they lived longer than 3 months after admission. Crude patient survival rate in the transplant group was higher than that in the nontransplant group (*p* = 0.003; Figure 2A). In the nontransplant group, patients with a high MELD score (≥30) (days 1, 7, or 14 after admission) had poorer outcomes than those with an MELD score of <30 (days 1, 7, or 14 after admission; *p* = 0.024; Figure 2B).

Crude patient survival for patients with DM was lower than for those without DM (*p* = 0.030; Figure 2C), and the same trend was observed in nontransplant patients with DM (Figure 2D). Compared with non-DM patients, patients with DM were associated with more hyperlipidemia (4/13 (30.8%) vs. 1/51 (2.0%), *p* = 0.005), more hepatic encephalopathy D-1 (4/13 (30.8%) vs. 3/51 (5.9%), *p* = 0.028), lower platelet count D-14 (90.3 ± 55.8 vs. 132.6 ± 71.3 × 10^3^/μL, *p* = 0.037), and higher MELD score D-7 (37.3 ± 21.1 vs. 27.7 ± 13.4, *p* = 0.046).

### 3.3. Univariable Risk Factor Analysis of Overall Survival

Table 3 shows that older age; presence of DM; increased lactate D-1; increased INR D-7 and D-14; presence of encephalopathy D-14; increased sodium level D-14; increased MELD score D-1, D-7, and D-14; and increased AARC score D-14 were risk factors (hazard ratio (HR) >1) associated with poorer patient survival in univariable analysis. By contrast, liver transplant was a protective factor with an HR of 0.14 (95% confidence interval, 0.03–0.62).

**Figure 3:**
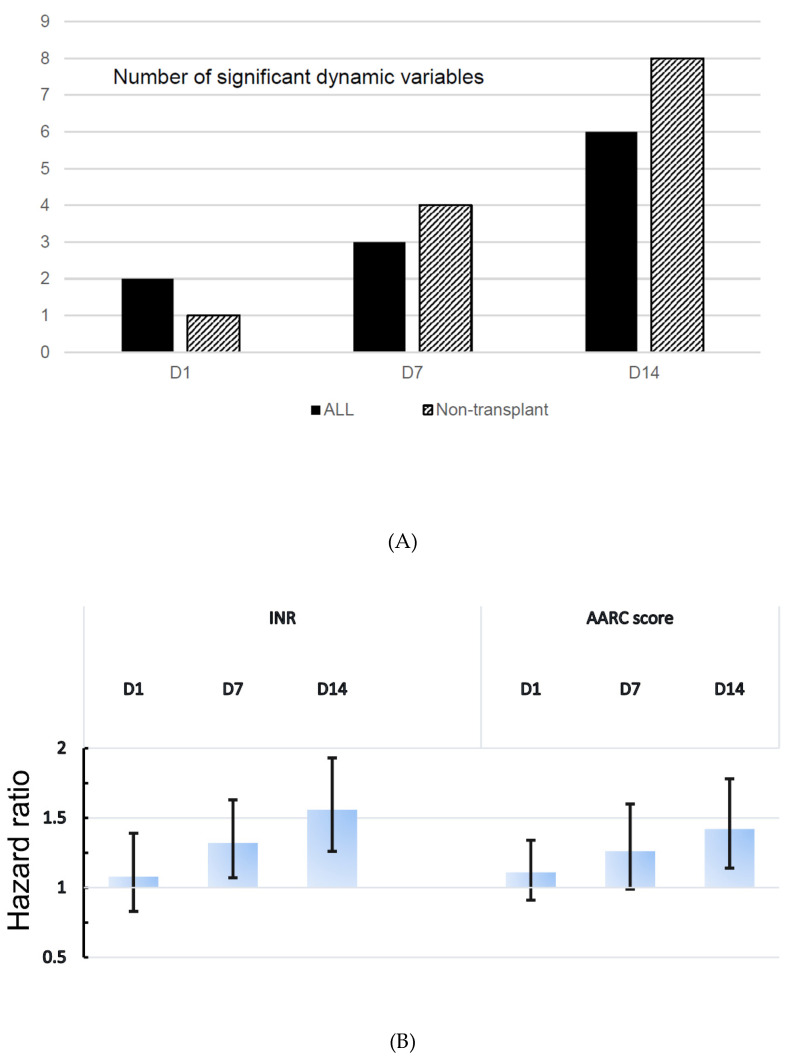
Dynamic prognostication. (**A**) Histogram showing the number of significant dynamic variables (at three time points (days 1, 7, and 14) after admission) associated with poorer patient survival in the nontransplant group and all patients with acute-on-chronic liver failure. (**B**) Trend of hazard ratios with 95% confidence interval for INR and AARC score at each time point. INR, international normalized ratio; AARC, APASL ACLF Research Consortium (ACLF, acute-on-chronic liver failure, APASL, the Asian Pacific Association for the Study of the Liver).

Among 18 dynamic variables (D-1, D-7, and D-14), nine were found to be nonsignificant at all three time points in univariable Cox analysis. Figure 3 shows the trend of dynamic prognostication. The number of significant dynamic variables was increased with time (Figure 3A). HR trends of the two most significant predictive variables (INR and AARC score) for each time point are shown in Figure 3B. Both risks increased in size and became significant after 1 week.

In the nontransplant group, old age; cirrhosis; massive ascites; higher INR (D-7 and D-14); presence of encephalopathy D-14; low platelet count; high ammonia, creatinine, and sodium D-14; high MELD scores (D-1, D-7, and D-14); and high AARC scores (D-7 and D-14) were risk factors associated with inferior survival in univariable analysis. In multivariable analysis, INR D-14 remained a statistically robust risk factor (HR 2.36 (1.15–4.81)) associated with inferior patient survival (Table 3).

### 3.4. Dynamic and Multivariable Risk Factor Analysis of Overall Survival

Because the dynamic variables were repeatedly measured at three time points in a small cohort, we performed initial multivariable Cox model analyses with backward selection to find out the most important dynamic variables among the three time points. INR D-14, sodium D-14, MELD score D-7, and AARC score D-14 were selected out in most models and were, therefore, chosen as the representative dynamic variables for further analysis.

Table 4 shows the adjusted risk factors associated with overall survival using Cox model analysis. In model 1, which included all selected dynamic variables, INR D-14 and AARC score D-14 were variables with a *p* value < 0.1. In multivariable analysis with backward selection (model 2), AARC score D-14 (adjusted HR, 1.66 (1.10–2.50)), and INR D-14 (adjusted HR, 1.61 (1.09–2.38)) were significant risk factors associated with inferior patient survival and liver transplant was a protective factor (adjusted HR, 0.04 (0.01–0.24)). In the nontransplant group, AARC score D-14 was a significant risk factor associated with inferior patient survival (HR, 2.12 (1.47–3.06)).

In summary, significant prognostic factors were established at day 14 after admission, and the period between days 7 and 14 was considered dynamically critical for therapeutic interventions to potentially reverse the prognosis.

## 4. Discussion

Our study had four main findings. First, 17.5% waitlisted adult patients met the ACLF diagnostic criteria, and most (87.5%) were HBV carriers. Second, although the MELD and AARC scores were similar between the transplant and nontransplant groups, patients with ACLF who received a liver transplant had poorer clinical and laboratory profiles. Third, almost all patients with ACLF received a live donor liver transplant and had superior survival than patients with ACLF without transplant. Irrespective of whether the patients received the transplant, 3-month survival after admission was critical in determining long-term outcome. Fourth, although multiple factors (particularly those at day 14) were associated with survival in univariable analysis, in multivariable analysis, AARC score at day 14 was associated with poor survival in the nontransplant group and all patients with ACLF.

Ideally, patients with ACLF whose livers have limited capacity for self-recovery should be transplanted in a timely manner to maximize transplant efficiency. In a pooled meta-analysis study with a large sample size from across the globe, liver transplant provided more survival benefit in patients with ACLF in earlier stages than in later stages [14,15]. In Western societies where deceased donors were the main organ source, allocation policies may not favor patients with ACLF and they are at a mortality disadvantage in the MELD-based system [16,17]. In our cohort of hospitalized patients, almost all liver grafts for transplant were derived from live donors with favorable short-term and long-term (3-year) overall survival. Live-donor transplant, therefore, is feasible when patients with ACLF are disadvantaged by the MELD-based organ allocation policy. Moreover, efforts in aggressive support care aimed at “downstaging” the severity score for ACLF can enhance posttransplant survival [18]. Plasma exchange has been shown to reverse organ failure and ameliorate the development of new organ failures and complications in patients with HBV-related ACLF [19,20]. Worsened INR or AARC score at day 14 after admission in our study suggests proceeding to fast-track liver transplant, or to plasmapheresis. In summary, expeditious decisions and implications for liver transplant [21], together with continuous downstaging efforts before surgery, are necessary to achieve favorable posttransplant outcomes in patients with ACLF.

**Figure 4:**
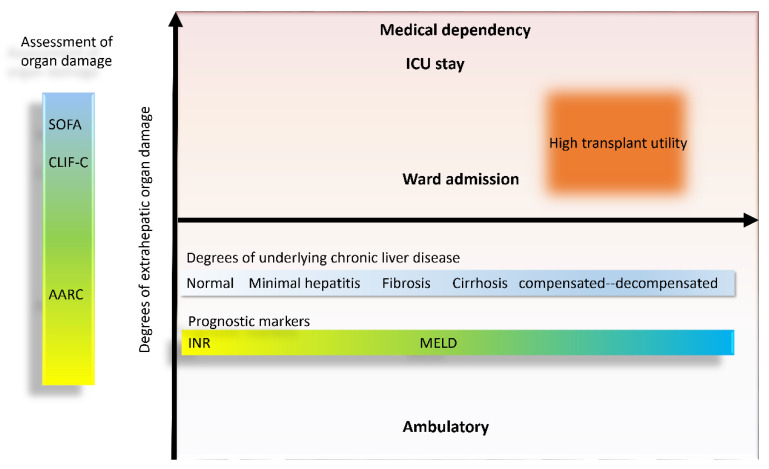
Proposed dynamic model of prognostication and transplant utility in acute-on-chronic liver failure. CLIF-C, European Foundation for the Study of Chronic Liver Failure; ICU, intensive care unit; INR, international normalized ratio; MELD, Model for End-stage Liver Disease; SOFA, Sequential Organ Failure Assessment.

The course of ACLF is highly dynamic and varies in stimulus strength and duration of acute triggers and liver functional reserve [1,2,22]. Therefore, a dynamic model to distinguish between those who will not survive without transplant and those who will recover with their own liver is a challenge [23]. Studies on comparisons between prognostic scores have been actively performed [24,25]. The MELD with serum sodium level (MELD-Na) score and scores based on the number of failing organs provide accurate prognostication for individual ACLF patients [3]. Studies specific to dynamic prognostication suggested that the score changes in a short period between day 3 and day 7 ***after diagnosis*** were correlated with prognosis and showed an indication for urgent liver transplantation [8,10]. Gustot et al. concluded that if the patient stays at, or progresses to, final grade 2 or 3 ACLF at day 3–7 after diagnosis of ACLF (based on CLIF-C ACLF criteria), defined as severe early course of ACLF, then prognosis is poor without an emergency liver transplantation and this assessment can provide a rational basis for intensive care discontinuation owing to futility [8]. Although our study results did not formulate practical recommendations about specific timing for switching to palliative care, the dynamic relationship between liver damage (acute and chronic), extrahepatic organ damage, severity assessment tools, and transplant utility can be illustrated in Figure 4. Patients with advanced liver damage and limited extrahepatic organ failures benefited most from a liver transplant, while those with severe extrahepatic multi-organ failures may not recover by just a liver transplant and succumb more to prognostic markers designated for the critically ill needing intensive care. Besides, less damage of ACLF was contributed by chronic liver background (MELD as the representative prognostic marker) and more by acute liver insult, and the more likely prognostic markers were consistent with that of acute liver failure (INR as the representative prognostic marker). The proposed dynamic model may impact future studies and clinical practice.

In our study, the number of significant dynamic variables was increased greatly between day 7 and day 14 ***after admission***. In addition, AARC score D-14 was a consistent prognostic factor in overall and nontransplant patient survival. Together, these results highlighted the golden period for therapeutic intervention to reverse the falls in prognosis within 2 weeks after admission. Timely high-intensity therapy with artificial liver support might benefit patients with ACLF on the waiting list [26]. Biologically, a pathway through interleukin-22 signal transducer and activator of transcription 3 was shown to promote tissue regeneration in ACLF [27]. Dynamic variables of AARC score and INR at day 14 after admission in our study might hint at the success of liver repair in ACLF and further studies are warranted.

Study limitations included selection bias, small sample size, retrospective design, and external application in all ACLFs with diverse combinations of etiologies. The lack of well-documented culture-proof bacterial infection precluded accurate ACLF diagnosis based on EASL-CLIF criteria, the start date of diagnostic confirmation, and then further analysis by sub-classification of severity grade. However, most patients were AARC-ACLF grade II (AARC score 8–10) in our cohort, which might suggest a translation to at least CLIF-C ACLF 1 or 2.

In conclusion, AARC score at day 14 was an independent prognostic factor associated with overall survival in patients with ACLF. Transplantation offers favorable outcomes to critically ill patients with ACLF and living donor liver transplantation shortens the waiting time.

## Figures and Tables

**Table 1:** Characteristics of patients with acute-on-chronic liver failure (ACLF) (clinical (A) and laboratory (B) profiles).

**(A)**					
**Variables**		**All (*n* = 64)**	**Without Transplant (*n* = 40)**	**With Transplant (*n* = 24)**	***p* Value**
Hospital mortality (*n*, %)		20 (31.3)	18 (45.0)	2 (8.3)	0.002
Age (mean years, SD)		53.5 (9.9)	54.2 (11.3)	52.2 (7.7)	0.458
Body mass index (kg/m^2^) (mean, SD)		25.0 (4.3)	25.0 (4.9)	25.0 (3.4)	0.976
Male sex (*n*, %)		46 (71.8)	26 (65.0)	20 (83.3)	0.242
Referred * (*n*, %)		25 (39.1)	11 (27.5)	14 (58.3)	0.033
Hepatitis B virus (*n*, %)		56 (87.5)	32 (80.0)	24 (100.0)	0.038
Diabetes mellitus (*n*, %)		13 (20.3)	10 (25.0)	3 (12.5)	0.337
Chronic kidney disease (*n*, %)		5 (7.8)	1 (2.5)	4 (16.7)	0.065
Hypertension (*n*, %)		11 (17.1)	8 (20.0)	3 (12.5)	0.509
Coronary artery disease (*n*, %)		2 (3.1)	2 (5.0)	0(0)	0.521
Hyperlipidemia (*n*, %)		5 (7.8)	4 (10.0)	1 (4.1)	0.641
Autoimmune disease (*n*, %)		9 (14.1)	7 (17.5)	2 (8.3)	0.462
Cirrhosis (*n*, %)		27 (42.1)	4 (10.0)	23 (95.8)	<0.001
Ascites (*n*, %)					<0.001
Mild		33 (51.6)	30 (75.0)	3 (12.5)	
Moderate		12 (18.8)	4 (10.0)	8 (33.3)	
Massive		19 (29.7)	6 (15.0)	13 (54.2)	
Plasma exchange (*n*, %)		15 (23.4)	5 (12.5)	10 (41.7)	0.014
Mean waiting time * (day, range)		-	-	27 (3–99)	-
Encephalopathy (*n*, %)	D1	7 (10.9)	4 (10.0)	3 (12.5)	1.000
	D7	5 (7.8)	3 (7.5)	2 (8.3)	1.000
	D14	9 (14.1)	6 (15.0)	3 (12.5)	1.000
MELD score (mean, SD)	D1	26.3 (12.6)	24.6 (9.4)	26.1 (6.8)	0.500
	D7	29.6 (15.5)	27.9 (14.5)	29.5 (9.7)	0.652
	D14	30.2 (16.7)	28.1 (16.1)	30.5 (10.7)	0.525
AARC score (mean, SD)	D1	8.4 (2.3)	8.3 (2.3)	8.8 (2.0)	0.402
	D7	8.6 (1.9)	8.3 (1.9)	9.0 (1.9)	0.214
	D14	9.0 (2.2)	8.6 (2.3)	9.6 (1.9)	0.122
**(B)**					
**Variables** **(Mean, SD)**		**All (*n* = 64)**	**Without Transplant (*n* = 40)**	**With Transplant (*n* = 24)**	***p* Value**
Hemoglobin g/dL	D1	13.5 (2.5)	13.5 (2.6)	13.8 (2.5)	0.721
	D7	12.0 (2.3)	12.0 (2.3)	12.0 (2.3)	0.939
	D14	11.1 (2.1)	11.3 (2.3)	10.9 (2.2)	0.496
WBC 10^3^/μL	D1	8.2 (3.6)	7.9 (4.0)	8.9 (3.1)	0.317
	D7	8.2 (3.5)	7.6 (3.3)	9.4 (3.6)	0.049
	D14	8.6 (4.4)	7.8 (3.2)	10.0 (5.7)	0.057
Platelet 10^3^/μL	D1	151.9 (79.4)	166.4 (90.2)	128.9 (52.4)	0.070
	D7	129.9 (62.6)	139.1 (69.0)	115.5 (48.8)	0.151
	D14	124.3 (70.1)	145.3 (77.8)	91.3 (38.0)	0.002
INR	D1	2.3 (1.4)	2.4 (1.6)	2.4 (1.2)	0.947
	D7	2.3 (1.5)	2.3 (1.6)	2.5 (1.4)	0.522
	D14	2.4 (1.4)	2.3 (1.4)	2.6 (1.6)	0.511
AST U/L	D1	969.9 (944.8)	1026.9 (975.3)	893.9 (917.6)	0.600
	D7	469.3 (771.9)	550.0 (927.2)	339.6 (403.2)	0.309
	D14	113.4 (98.8)	124.8 (116.1)	97.0 (65.9)	0.305
ALT U/L	D1	1194.1 (1174.6)	1258.2 (1246.9)	1090.1 (1064.2)	0.585
	D7	556.3 (758.9)	630.6 (838.2)	438.8 (611.4)	0.336
	D14	155.0 (172.4)	165.1 (174.8)	139.2 (171.1)	0.569
Total bilirubin	D1	17.2 (11.0)	16.3 (11.1)	19.0 (11.2)	0.349
mg/dL	D7	21.1 (9.2)	20.3 (8.3)	22.5 (10.5)	0.358
	D14	23.6 (12.4)	22.5 (12.5)	25.6 (12.4)	0.341
Albumin g/dL	D1	3.3 (0.6)	3.4 (0.7)	3.2 (0.7)	0.252
	D7	3 (0.4)	3.0 (0.4)	3.1 (0.5)	0.501
	D14	3.1 (0.4)	2.9 (0.5)	3.2 (0.5)	0.062
BUN mg/dL	D1	16.0 (17.1)	17.5 (21.0)	14.2 (9.9)	0.544
	D7	21.8 (24.6)	19.6 (24.0)	25.3 (25.8)	0.400
	D14	24.4 (24.4)	19.9 (20.7)	30.3 (27.9)	0.118
Creatinine mg/dL	D1	1.2 (1.1)	1.2 (0.8)	1.4 (1.5)	0.559
	D7	1.6 (1.8)	1.5 (1.6)	2.0 (2.1)	0.277
	D14	1.4 (1.4)	1.1 (0.6)	2.1 (2.0)	0.009
Sodium mmol/L	D1	133.3 (5.2)	133.52 (4.8)	133.1 (5.9)	0.771
	D7	134.8 (6.2)	134.9 (5.2)	134.6 (7.9)	0.825
	D14	135.5 (6.6)	136.3 (6.3)	134.4 (7.3)	0.314
CRP mg/L	D1	2.0 (2.5)	2.6 (2.9)	0.9 (0.9)	0.196
	D7	1.9 (2.3)	2.4 (2.7)	0.9 (0.9)	0.275
	D14	1.8 (1.8)	2.26 (2.2)	1.2 (0.8)	0.216
pH	D1	7.42 (0.07)	7.39 (0.1)	7.43 (0.05)	0.278
	D7	7.44 (0.04)	7.44 (0.03)	7.44 (0.05)	1.000
	D14	7.44 (0.05)	7.44 (0.05)	7.45 (0.04)	0.760
Ammonia μmol/L	D1	99.4 (84.1)	103.7 (95.3)	93.3 (66.1)	0.644
	D7	77.4 (36.0)	74.2 (25.4)	82.3 (48.1)	0.399
	D14	74.2 (34.9)	73.3 (28.4)	75.6 (42.9)	0.810
Lactate mmol/L	D1	4.7 (8.1)	5.8 (10.1)	2.8 (1.3)	0.420
	D7	2.1 (1.7)	2.3 (1.9)	1.9 (1.3)	0.681
	D14	2.5 (1.6)	2.5 (1.7)	2.5 (1.6)	0.965

AARC: APASL ACLF Research Consortium (APASL, the Asian Pacific Association for the Study of the Liver); AST: aspartate aminotransaminase; ALT: alanine aminotransferase; BUN: blood urea nitrogen; CI: confidence interval; CRP: C-reactive protein; INR: international normalized ratio; MELD: Model for End-stage Liver Disease; WBC: white blood cell count; * Mean waiting time: time from the day of registration on the waiting list to the day of liver transplantation; Referred: received treatment at other hospitals before admission.

**Table 2:** Characteristics of transplant-free patients with acute-on-chronic liver failure (ACLF) (3-month survivors and non-survivors).

Variables		Survivors (*n* = 22)	Non-survivors (*n* = 18)	*p* Value
Age (mean years, SD)		50.0 (9.8)	59.7 (10.9)	0.006
Body mass index (kg/m^2^) (mean, SD)		24.8 (4.5)	25.4 (5.4)	0.691
Male (*n*, %)		13 (59.0)	13 (72.2)	0.318
Hepatitis B virus (*n*, %)		15 (68.2)	18 (100)	0.011
Diabetes mellitus (*n*, %)		4 (18.1)	6 (33.3)	0.282
Chronic kidney disease (*n*, %)		0 (0)	1 (5.6)	0.436
Hypertension (*n*, %)		3 (13.6)	5 (27.8)	0.261
Coronary artery disease (*n*, %)		1 (4.5)	1 (5.6)	1.000
Hyperlipidemia (*n*, %)		3 (13.6)	1 (5.6)	0.618
Autoimmune disease (*n*, %)		4 (18.1)	3 (16.7)	1.000
Cirrhosis (*n*, %)		4 (18.1)	9 (50.0)	0.039
Ascites (*n*, %)				0.006
Mild		20 (90.9)	10 (55.6)	
Moderate		2 (9.1)	2 (11.1)	
Massive		0 (0)	6 (33.3)	
Plasma exchange (*n*, %)		0 (0)	5 (27.8)	0.011
Encephalopathy (*n*, %)	D1	1 (4.5)	3 (16.7)	0.300
	D7	1 (4.5)	2 (11.1)	0.562
	D14	1 (4.5)	5 (27.8)	0.065
Platelet (10^3^/uL)	D1	182.1 (90.7)	144.9 (87.8)	0.212
(mean, SD)	D7	159.5 (65.3)	110.8 (65.7)	0.030
	D14	178.6 (79.4)	99.5 (47.4)	<0.001
INR (mean, SD)	D1	2.23 (1.84)	2.57 (1.35)	0.516
	D7	1.69 (0.69)	3.02 (2.14)	0.011
	D14	1.61 (0.71)	3.31 (1.46)	<0.001
Ammonia (μmol/L)	D1	119.5 (116.8)	82.7 (51.9)	0.220
(mean, SD)	D7	73.3 (20.8)	75.4 (31.5)	0.826
	D14	65.1 (18.6)	85.8 (36.3)	0.039
Creatinine (mg/dL)	D1	1.2 (0.9)	1.2 (0.6)	0.755
(mean, SD)	D7	1.5 (1.9)	1.5 (1.1)	0.939
	D14	0.9 (0.2)	1.4 (0.7)	0.006
Sodium (mmol/L)	D1	132.6 (4.1)	134.6 (5.5)	0.218
(mean, SD)	D7	134.7 (4.8)	135.3 (5.9)	0.739
	D14	134.2 (4.9)	139.0 (7.2)	0.025
MELD score (mean, SD)	D1	23.7 (10.3)	25.7 (8.1)	0.508
	D7	22.8 (7.4)	34.2 (18.4)	0.014
	D14	20.9 (8.4)	37.1 (19.0)	0.002
AARC score (mean, SD)	D1	7.8 (2.1)	8.8 (2.7)	0.181
	D7	7.8 (1.7)	9.2 (2.0)	0.028
	D14	7.5 (1.7)	10.3 (2.2)	<0.001

AARC: APASL ACLF Research Consortium (APASL, the Asian Pacific Association for the Study of the Liver); BUN: blood urine nitrogen; CI: confidence interval; INR: international normalized ratio; MELD: Model for End-Stage Liver Disease.

**Table 3:** Univariable risk factors associated with overall survival.

		All		Nontransplant	
		HR (95% Cl)	*p* Value	HR (95% Cl)	*p* Value
Age		1.08 (1.03–1.14)	0.003	1.06 (1.01–1.11)	0.015
Diabetes mellitus		2.67 (1.06–6.71)	0.037	1.91 (0.71–5.09)	0.201
Cirrhosis		0.92 (0.38–2.23)	0.861	2.88 (1.13–7.34)	0.026
Ascites			0.800		0.005
Mild (reference)		-	-	-	-
Moderate		0.80 (0.22–2.90)	0.731	1.97 (0.43–9.02)	0.383
Massive		1.24 (0.47–3.26)	0.661	5.92 (2.05–17.13)	0.001
Liver transplant		0.14 (0.03–0.62)	<0.001	-	-
Encephalopathy	D1	1.89 (0.55–6.48)	0.313	3.29 (0.94–11.55)	0.064
	D7	1.49 (0.34–6.47)	0.597	2.28 (0.51–19.08)	0.279
	D14	3.03 (1.08–8.53)	0.036	4.55 (1.55–13.33)	0.006
Platelet	D1	1.00 (0.99–1.00)	0.292	1.00 (0.99–1.00)	0.175
	D7	0.99 (0.98–1.00)	0.037	0.99 (0.98–1.00)	0.024
	D14	0.99 (0.98–1.00)	0.035	0.99 (0.98–1.00)	0.004
INR	D1	1.08 (0.83–1.39)	0.575	1.09 (0.87–1.36)	0.462
	D7	1.32 (1.07–1.63)	0.008	1.35 (1.11–1.63)	0.002
	D14	1.56 (1.26–1.93)	<0.001	2.29 (1.60–3.27)	<0.001
Sodium	D1	1.07 (0.98–1.18)	0.128	1.09 (0.98–1.21)	0.109
	D7	1.02 (0.95–1.09)	0.659	1.02 (0.93–1.13)	0.638
	D14	1.10 (1.03–1.18)	0.004	1.12 (1.04–1.21)	0.004
MELD score	D1	1.04 (1.00–1.00)	0.013	1.04 (1.01–1.07)	0.022
	D7	1.07 (1.03–1.11)	<0.001	1.10 (1.04–1.16)	<0.001
	D14	1.08 (1.04–1.11)	<0.001	1.12 (1.06–1.20)	<0.001
AARC score	D1	1.11 (0.91–1.34)	0.309	1.18 (0.97–1.43)	0.094
	D7	1.26 (0.99–1.60)	0.065	1.45 (1.10–1.90)	0.008
	D14	1.42 (1.14–1.78)	0.002	2.05 (1.48–2.82)	<0.001
Lactate	D1	1.07 (1.00–1.14)	0.033	1.06 (0.99–1.12)	0.081
	D7	1.14 (0.82–1.57)	0.435	1.07 (0.77–1.49)	0.684
	D14	1.42 (0.90–2.24)	0.128	11.25 (0.45–280.87)	0.140
Ammonia	D1	1.00 (0.99–1.01)	0.595	0.99 (0.98–1.01)	0.363
	D7	1.00 (0.99–1.02)	0.774	1.01 (0.99–1.03)	0.561
	D14	1.01 (1.00–1.02)	0.113	1.03 (1.01–1.06)	0.003
Creatinine	D1	1.16 (0.90–1.50)	0.242	1.08 (0.62–1.88)	0.789
	D7	1.02 (0.82–1.28)	0.836	1.01 (0.76–1.34)	0.939
	D14	1.05 (0.79–1.39)	0.756	3.06 (1.50–6.26)	0.002

AARC: APASL ACLF Research Consortium (ACLF, acute-on-chronic liver failure, APASL, the Asian Pacific Association for the Study of the Liver); CI: confidence interval; HR: hazard ratio; INR: international normalized ratio; MELD: Model for End-stage Liver Disease. Nonsignificant factors: albumin, body mass index, blood urea nitrogen, chronic kidney disease, coronary artery disease, hemodialysis, hepatitis B virus, hypertension, male sex, and white blood cell count.

**Table 4:** Adjusted risk factors associated with overall survival.

		All				Nontransplant			
Variables		Model 1 HR (95% CI)	*p* Value	Model 2 * HR (95% CI)	*p* Value	Model 1 HR (95% CI)	*p* Value	Model 2 * HR (95% CI)	*p* Value
Age		1.03 (0.96–1.11)	0.377	-	-	1.02 (0.95–1.09)	0.647	-	-
DM		1.06 (0.26–4.36)	0.934	-	-	0.60 (0.11–3.15)	0.545	-	-
Liver transplant		0.05 (0.01–0.34)	0.002	0.04 (0.01–0.24)	<0.001	-	-	-	-
INR	D14	1.66 (1.08–2.55)	0.021	1.61 (1.09–2.38)	0.017	1.62 (0.95–2.74)	0.075	-	-
Sodium	D14	1.06 (0.97–1.16)	0.213	-	-	1.08 (0.97–1.19)	0.166	-	-
MELD score	D7	0.98 (0.89–1.08)	0.630	-	-	0.97 (0.88–1.07)	0.576	-	-
AARC score	D14	1.57 (0.98–2.52)	0.062	1.66 (1.10–2.50)	0.016	1.74 (1.02–2.95)	0.040	2.12 (1.47–3.06)	<0.001

* Cox model analysis with backward selection. AARC: APASL ACLF Research Consortium (ACLF, acute-on-chronic liver failure, APASL, the Asian Pacific Association for the Study of the Liver); CI: confidence interval; DM: Diabetes mellitus; HR: hazard ratio; INR: international normalized ratio; MELD: Model for End-stage Liver Disease.

## Data Availability

The datasets used and analyzed during the current study are available from the corresponding author upon reasonable request.

## Abbreviations

AARC	APASL ACLF Research Consortium
ACLF	acute-on-chronic liver failure
APASL	the Asian Pacific Association for the Study of the Liver
CI	confidence interval
DM	diabetes mellitus
EASL-CLIF	the European Association for the Study of the Liver–chronic liver failure
HBV	hepatitis B
HCV	hepatitis C
HR	hazard ratio
INR	international normalized ratio
MELD	Model for End-Stage Liver Disease
NASCELD	the North American Consortium for the Study of End-Stage Liver Disease
